# Accuracy of glomerular filtration rate estimation based on creatinine and cystatin C for monitoring moderate chronic kidney disease in adults: prospective, longitudinal cohort study

**DOI:** 10.1136/bmj-2025-085005

**Published:** 2026-03-19

**Authors:** Katie Scandrett, Alice J Sitch, Jonathan Barratt, Elizabeth A Brettell, Paul Cockwell, R Neil Dalton, Jonathan James Deeks, Gillian Eaglestone, Philip A Kalra, Kamlesh Khunti, Fiona C Loud, Ryan Ottridge, Tracy Pellatt-Higgins, Aisling Potter, Ceri Rowe, Claire C Sharpe, Bethany Shinkins, Alison Smith, Paul E Stevens, Andrew J Sutton, Maarten W Taal, Edmund J Lamb

**Affiliations:** 1 Department of Applied Health Sciences, University of Birmingham, Birmingham, UK; 2 National Institute for Health and Care Research (NIHR) Birmingham Biomedical Research Centre, Birmingham, UK; 3 Department of Cardiovascular Sciences, University of Leicester, Leicester, UK; 4 Birmingham Clinical Trials Unit, Department of Applied Health Sciences, University of Birmingham, Birmingham, UK; 5 Renal Medicine, Queen Elizabeth Hospital Birmingham, Birmingham, UK; 6 Institute of Inflammation and Ageing, University of Birmingham, Birmingham, UK; 7 Department of Paediatrics, Guy’s and St Thomas’s NHS Foundation Trust, London, UK; 8 Kent Kidney Care Centre, East Kent Hospitals University NHS Foundation Trust, Canterbury, Kent, UK; 9 Department of Renal Medicine, Salford Royal Hospital, Northern Care Alliance NHS Foundation Trust, Salford, UK; 10 Diabetes Research Centre, University of Leicester, Leicester, UK; 11 Kidney Care UK, Alton, UK; 12 Centre for Health Services Studies, University of Kent, Canterbury, UK; 13 Clinical Biochemistry, East Kent Hospitals University NHS Foundation Trust, Canterbury CT1 3NG, UK; 14 School of Medicine, University of Nottingham, Nottingham, UK; 15 Academic Unit of Health Economics, Leeds Institute of Health Sciences, University of Leeds, Leeds, UK; 16 Warwick Medical School, University of Warwick, Coventry, UK; 17 Department of Renal Medicine, University Hospitals of Derby and Burton NHS Foundation Trust, Derby, UK; 18 Division of Medical Sciences and Graduate Entry Medicine, University of Nottingham, Nottingham, UK

## Abstract

**Objectives:**

To provide evidence on the longitudinal accuracy of glomerular filtration rate (GFR) estimating equations that include creatinine and cystatin C to monitor patients with moderate chronic kidney disease.

**Design:**

Prospective, longitudinal, cohort study.

**Setting:**

Primary, secondary, and tertiary care across six centres in England, 1 April 2014 to 31 December 2017.

**Participants:**

1229 adults (≥18 years) with moderate chronic kidney disease (creatinine estimated GFR of 30-59 mL/min/1.73 m^2^ for at least three successive months before recruitment).

**Main outcome measures:**

Ability of estimating equations to monitor GFR over three years, with slope deviations from reference measured GFR (iohexol clearance) within ±3 mL/min/1.73 m^2^/year indicating agreement. Ability of GFR estimating equations to detect disease progression (ie, a reduction in measured GFR of ≥25% with a reduction in disease category).

**Results:**

After three years, 875 participants had measured and estimated GFR data recorded at the start and end of the study and comprised the study cohort. Median measured GFR decreased from 48.1 mL/min/1.73 m^2 ^at baseline to 43.6 mL/min/1.73 m^2 ^at three years. GFR was estimated with the Chronic Kidney Disease Epidemiology Collaboration (CKD-EPI) and European Kidney Function Consortium (EKFC) estimating equations. Median change in measured GFR exceeded median change in estimated GFR for all equations. All equations achieved agreement with change in measured GFR in >72.5% of participants. Dual biomarker equations showed better agreement with change in measured GFR (CKD-EPI_creatinine-cystatin_ 78.6% of individuals, 95% confidence interval 75.8% to 81.3%; CKD-EPI(2021)_creatinine-cystatin_ 78.1%, 75.2% to 80.8%; and EKFC_creatinine-cystatin_ 80.2%, 77.4% to 82.8%) than CKD-EPI_creatinine_ (73.1%, 70.1% to 76.1%) (all P<0.001). Progression of kidney disease was seen in 139 (15.9%) individuals. All GFR equations had poor sensitivity (<54.1%) but good specificity (>90.4%) for identifying progression of chronic kidney disease.

**Conclusions:**

Underestimation of the reduction in GFR by estimated GFR requires further investigation. Equations that included both creatinine and cystatin C more accurately monitored change in measured GFR than equations based on one biomarker. Increased use of combined biomarker equations in clinical practice could improve disease monitoring and potentially clinical care.

**Study registration:**

ISRCTN registry ISRCTN42955626.

## Introduction

Chronic kidney disease is commonly detected and monitored by glomerular filtration rate (GFR) or albuminuria, or both. Absolute GFR level and change in GFR level affect many clinical decisions in the management of chronic kidney disease.^1^ GFR can be estimated with a variety of equations. Historically, these equations were mainly based on measurement of serum creatinine concentration, with adjustments for age and sex. More recently, equations have been described that incorporate serum cystatin C concentration, a protein biomarker of kidney function. Most studies have focused on the relative accuracy of estimated GFR equations to reflect measured GFR at one point in time. Few prospective studies exist of the ability of GFR estimating equations, in particular those incorporating cystatin C, to monitor and detect change in GFR and progression of kidney disease.

No consistent definition of progression of kidney disease exists. Research studies and clinical practice often use different criteria. Kidney Disease: Improving Global Outcomes (KDIGO) defines progression of chronic kidney disease as a change to a higher disease category (eg, stage 3A (GFR 45-59 mL/min/1.73 m^2^) to stage 3B (GFR 30-44 mL/min/1.73 m^2^)), together with a decrease in GFR of ≥25% (eg, a reduction from 50 to 35 mL/min/1.73 m^2^) or an increase in albuminuria category.^2^ Rapid progression was defined as a sustained reduction in GFR of >5 mL/min/1.73 m^2^/year (eg, a reduction from 60 to <55 mL/min/1.73 m^2^ in one year).^2^ The National Institute for Health and Care Excellence (NICE) defines accelerated progression as either a sustained decrease in GFR of ≥25% and a change in GFR category within 12 months, or a sustained decrease in GFR of 15 mL/min/1.73 m^2^/year.^1^

In clinical practice, identifying progression of chronic kidney disease is challenging and confounded by the biological and measurement variability of both reference and estimated GFR.^3^ Prospective, large, longitudinal studies assessing the relative abilities of GFR estimating equations to detect reductions in underlying true GFR are lacking. A large retrospective study (3532 participants with chronic kidney disease followed for a mean of 2.6 years) examined the accuracy of GFR estimating equations compared with ^125^I-iothalamate measured GFR over time.^4^ The authors found that GFR estimating equations accurately reflected changes in measured GFR. This study, however, did not include estimated GFR data derived from cystatin C. In the present study, we assessed the ability of published estimates of GFR, including those incorporating cystatin C, to monitor changes in measured GFR over time and detect reductions in measured GFR consistent with disease progression, as defined by KDIGO.^2^

## Methods

In this prospective cohort study, we recruited individuals aged ≥18 years with stage 3 chronic kidney disease from six sites across England. Participants had estimated GFR measurements of 30-59 mL/min/1.73 m^2^ for at least three successive months before recruitment.^5^ The international chronic kidney disease staging system requires knowledge of both GFR and albuminuria to define stage: in this study, stage 3 chronic kidney disease refers to all individuals with a GFR value of 30-59 mL/min/1.73 m^2^, irrespective of albuminuria status. Patients were recruited from 1 April 2014 to 31 December 2017.

GFR was measured with an iohexol clearance method. At the same time, blood was taken for measurement of serum concentrations of creatinine and cystatin C, and a urine sample collected to measure the albumin to creatinine ratio.^5^ The measurement methods for serum cystatin C and creatinine, urine albumin to creatinine ratio, and iohexol measured GFR are described elsewhere.^6^ ^7^ GFR was estimated with the Chronic Kidney Disease Epidemiology Collaboration (CKD-EPI) and European Kidney Function Consortium (EKFC) estimating equations: CKD-EPI_creatinine_,^8^ CKD-EPI_cystatin_,^9^ CKD-EPI_creatinine-cystatin_,^9^ EKFC_creatinine_,^10^ EKFC_cystatin_,^11^ and EKFC_creatinine-cystatin_,^11^ and the 2021 revisions of the CKD-EPI equations^12^ (supplementary table 1). Participants were followed for three years, with measurements repeated at 36 months. GFR was also estimated at six monthly intervals in all participants (seven estimated GFR values in total). Some participants underwent additional measured GFR testing at one (n=215) and two (n=188) years (up to four measured GFR values in total) (supplementary fig 1). The supplementary file has more information about recruitment, inclusion and exclusion criteria, data collection, and sampling.

### Sample size and general statistical considerations

The original sample size calculation suggested that the study had 90% power to detect differences in the proportion of participants with ≥±3 mL/min/1.73 m^2^/year error and >80% power to detect differences in the proportion of patients with >±5%/year error with a sample size of 1300, allowing for 15-20% dropouts over three years. Revised sample size calculations suggested that with 875 evaluable participants, the study had >85% power to compare proportions of equations with ≥±3 mL/min/1.73 m^2^/year error over three years. A P value <0.05 was considered significant. Multiple comparisons were not formally adjusted for because the estimating equations are not independent.

### Statistical analyses

#### Primary analysis

In the primary analysis, we were interested in the ability of estimated GFR to reflect change (slope) in either direction in measured GFR over time at the individual patient level. For each participant, the difference between baseline and three year follow-up values was calculated for each estimated and measured GFR value. The estimated change per year (slope) was derived by averaging the change over time between baseline and the three year follow-up (equation 1):

- Measured GFR slope=(measured GFR at follow-up−measured GFR at baseline)÷(years between baseline and follow-up)
- Estimated GFR slope=(estimated GFR at follow-up−estimated GFR at baseline)÷(years between baseline and follow-up)

The rate of change was calculated based on actual calendar sampling date where the follow-up period was not exactly three years. 

The outcome of interest was the difference between the measured and estimated GFR slope, calculated by subtracting the observed measured GFR slope from the observed estimated GFR slope. If this difference between estimated and measured GFR slope was ≥3 mL/min/1.73 m^2^/year or ≤−3 mL/min/1.73 m^2^/year, the difference was considered a large error (referred to as ≥±3 mL/min/1.73 m^2^/year from here onwards) (supplementary material has more details).^4^

The percentage of participants without a large error (ie, those showing agreement) when comparing the slope values between each estimated GFR equation and measured GFR, with corresponding exact binomial 95% confidence intervals (CIs), was calculated. Since the CKD-EPI_creatinine_ equation is recommended for clinical use in England,^1^ we conducted pairwise comparisons of the percentages of participants without a large error for all GFR estimating equations against the original versions of the CKD-EPI equations with McNemar’s test for paired data.^8^ ^9^ In response to a peer reviewer’s suggestion, reanalysis of the data stratified by sex was included. Patient's data for sex were from assigned sex rather than self-reported gender. All analyses were conducted with Stata version 18.0 or 19.0.

#### Sensitivity analyses of the primary analysis

Two different approaches were investigated for calculating the change in estimated and measured GFR values as sensitivity analyses.^4^ These sensitivity analyses were conducted for the three original CKD-EPI equations,^8^ ^9^ with the criterion of ≥±3 mL/min/1.73 m^2^/year difference between the estimated and measured GFR slopes indicating a large error.

In the first sensitivity analysis, the change in estimated GFR values (with up to seven estimated GFR measurements for each individual) was calculated by fitting a linear regression model for each individual.^4^ These slope values were compared with the observed measured GFR slope, calculated as previously described. In the second sensitivity analysis, the slope values calculated with the linear regression model for each estimated GFR were then compared with the estimated change in measured GFR from a multilevel linear regression model (estimated slopes for each individual were derived from one mixed effects model with up to four measured GFR values for each individual).

#### Secondary analyses

The above analysis was repeated with a criterion of >5%/year or <−5%/year difference in the slope between estimated and measured GFR indicating a large error (referred to as >±5%/year from here onwards).^4^ Estimated and measured GFR slope values (equation 1) were divided by their respective baseline values to determine the percentage change relative to the baseline value.

For each participant, whether or not their change in measured GFR (reference test) and estimated GFR values (index tests) were reduced by ≥25% in combination with a progression in disease category was calculated over the three years (supplementary table 2). These criteria were chosen to align with the internationally agreed definition of progression of chronic kidney disease.^2^ The numbers of participants fulfilling these criteria, as assessed by estimated or measured GFR values, were compared to calculate the sensitivity, specificity, positive predictive value, and negative predictive value for each of the equations. Exact binomial 95% CIs were calculated for each metric.

Median differences between estimated and measured GFR values were calculated at baseline and at the three year follow-up to provide measures of bias. This same analysis was repeated after grouping the data by those who did and did not show disease progression. The accuracy of the GFR estimating equations compared with measured GFR values was calculated at baseline and at the three year follow-up by establishing the proportion of GFR estimates within 30% (P_30_) of iohexol measured GFR, with corresponding 95% CIs.^13^

### Patient and public involvement

A patient representing Kidney Care UK (www.kidneycareuk.org/) was a member of the full study project management group and another patient representative was a member of the study steering committee, which met about every six months. Both individuals provided expert patient input, recommendations on patient involvement, and patient representation on study newsletters. Retention in the study was encouraged through newsletters and sending final appointment reminder letters. Participant information leaflets were prepared in collaboration with the patient representatives and were circulated for comment to patient groups at the recruiting units and to the Research Design Service South East public patient involvement group. Recruitment and retention strategies were adjusted to meet the needs of specific ethnic minority groups, including the production of translated material and the use of translators where required for non-English speaking participants.

## Results

Of the 1229 participants recruited to the study, 1205 and 1180 participants had evaluable estimated and measured GFR values, respectively, at baseline, with 1167 (95.0%) having both estimated and measured GFR values recorded. At 36 months, 976 participants remained in the study, of whom 875 (71.2%) had evaluable estimated and measured GFR values at both baseline and 36 months. Overall, 253 participants withdrew from the study: consent was withdrawn by 112 participants, 79 were lost to follow-up, and 62 died (supplementary figure 1). The characteristics of the cohort monitored were broadly similar to the larger baseline cohort (n=1167) from which the participants were drawn (supplementary table 3). Baseline accuracy of the GFR estimating equations, as measured by P_30_ in the 875 individuals included in the monitoring study, was similar to that of the overall cohort (n=1167),^7^ but was slightly reduced at the three year follow-up (supplementary table 4).

Table 1 shows the characteristics of the 875 participants at baseline (supplementary table 3 has more baseline characteristics). Median age was 67.1 years and 505 (57.7%) participants were men. Most participants were white (n=773, 88.3%) and the most common comorbidity was diabetes (n=220, 25.1%). At baseline, 38 (4.3%) participants in the cohort had measured GFR <30 mL/min/1.73 m^2^ and 168 (19.2%) had measured GFR ≥60 mL/min/1.73 m^2^. Of the 875 participants, seven (0.8%) had kidney failure (measured GFR <15 mL/min/1.73 m^2^) at the three year follow-up.

**Table 1:** Baseline characteristics of study participants

Characteristic	All participants with measured and estimated GFR at baseline and 3 year follow-up (n=875)
Age (years)	67.1 (58.1-73.6)
No of men:women	505:370
Ethnic group (No (%)):	
White	773 (88.3)
Black	36 (4.1)
South Asian	46 (5.3)
Other*	20 (2.3)
Height (cm)	170 (163-176)
Weight (kg)	84.7 (73-97.2)
Body surface area (m^2^)	1.96 (1.81-2.11)
Body mass index	29.0 (25.7-33.4)
Urine albumin concentration (mg/mmol) (No (%)):	
<3	375 (42.9)
3-30	295 (33.7)
>30	195 (22.3)
Missing data	10 (1.1)
Serum creatinine (µmol/L)	128 (106-151)
Serum cystatin C (mg/L)	1.48 (1.25-1.76)
Measured GFR (mL/min/1.73 m^2^)	48.1 (40.2-57.2)
Estimated GFR (mL/min/1.73 m^2^):	
CKD-EPI_creatinine_	45.7 (37.7-54.2)
CKD-EPI_cystatin_	43.6 (35.0-54.3)
CKD-EPI_creatinine-cystatin_	43.4 (35.9-53.3)
CKD-EPI(2021)_creatinine_	48.3 (39.5-57.2)
CKD-EPI(2021)_creatinine-cystatin_	43.3 (35.9-53.2)
EKFC_creatinine_	43.8 (36.5-51.5)
EKFC_cystatin_	47.2 (39.1-57.8)
EKFC_creatinine-cystatin_	45.6 (38.2-54.3)
Chronic kidney disease GFR category at baseline based on measured GFR (No (%)):	
Stage 1	5 (0.6)
Stage 2	163 (18.6)
Stage 3A	366 (41.8)
Stage 3B	303 (34.6)
Stage 4	38 (4.3)
Stage 5	0

Data are median (IQR) unless indicated otherwise.

* Includes participants with ethnic background other than white, South Asian or black, as well as three individuals where data were not recorded.

GFR=glomerular filtration rate; CKD-EPI=Chronic Kidney Disease Epidemiology Collaboration estimating equation; EKFC=European Kidney Function Consortium GFR estimating equation.

Median measured GFR decreased from 48.1 mL/min/1.73 m^2 ^at baseline to 43.6 mL/min/1.73 m^2^ (decline of 4.5) at the three year follow-up. The median change in measured GFR exceeded the median change in estimated GFR over the three year study period for all equations: for example, the equivalent change for the CKD-EPI_creatinine_ equation was 45.7 to 42.0 mL/min/1.73 m^2^ (decline of 3.7) (supplementary table 4). The extent of underestimation of change is best shown among the 139 (15.9%) individuals who had disease progression. Participants with disease progression showed a greater underestimation of measured GFR by estimated GFR at baseline than participants with no disease progression, but over the study period underestimated the reduction in measured GFR. For example, median reduction in measured GFR was 16.4 mL/min/1.73 m^2^ compared with 12.2 mL/min/1.73 m^2^ for CKD-EPI_creatinine_, with all other estimating equations showing a similar underestimate of the reduction in GFR (table 2). Consequently, we saw a decrease in the negative bias of estimated compared with measured GFR over time. Participants with disease progression were more likely to be men and to have diabetes and severe albuminuria than participants with no disease progression (supplementary table 5).

**Table 2:** Measured and estimated changes in glomerular filtration rate (GFR mL/min/1.73 m^2^) in participants with disease progression and in those who did not have disease progression (based on ≥25% reduction in measured GFR and reduction in disease category)

Measure or estimate of GFR	Disease progression (n=139)				No disease progression (n=736)		
	Baseline GFR	Follow-up GFR	Change (follow-up – baseline)		Baseline GFR	Follow-up GFR	Change (follow-up – baseline)
Measured GFR	47.7 (39.1-55.1)	29.6 (23.9-36.4)	−16.4 (−20.0 to −13.7)		48.4 (40.4-57.8)	46.3 (38.1-55.7)	−3.0 (−7.5 to 1.7)
Estimated GFR:							
CKD-EPI_creatinine_	43.1 (37.3-49.3)	29.1 (22.3-37.5)	−12.2 (−18.7 to −7.3)		46.6 (37.8-55.0)	44.4 (35.0-53.7)	−2.5 (−7.3 to 2.4)
CKD-EPI_cystatin_	39.0 (31.6-48.0)	27.5 (20.0-36.5)	−10.9 (−16.0 to −6.2)		44.6 (35.7-55.1)	43.0 (32.9-54.6)	−2.0 (−7.4 to 3.0)
CKD-EPI_creatinine-cystatin_	40.6 (34.0-48.0)	28.1 (21.6-35.2)	−11.1 (−17.1 to −7.6)		44.8 (36.3-54.5)	43.1 (33.4-53.6)	−2.4 (−6.9 to 2.6)
CKD-EPI(2021)_creatinine_	45.6 (39.1-51.7)	31.2 (24.0-40.2)	−12.8 (−19.6 to −7.7)		49.1 (39.9-57.7)	47.0 (37.1-56.6)	−2.5 (−7.6 to 2.7)
CKD-EPI(2021)_creatinine-cystatin_	43.0 (35.9-50.9)	30.2 (22.0-37.3)	−11.9 (−17.9 to−8.0)		47.0 (38.6-57.8)	45.8 (35.6-57.1)	−2.3 (−7.4 to 2.9)
EKFC_creatinine_	41.7 (36.2-47.6)	29.5 (22.1-36.4)	−11.2 (−17.2 to −6.9)		44.7 (36.5-52.0)	42.2 (33.8-50.6)	−2.7 (−6.9 to 1.8)
EKFC_cystatin_	43.1 (36.5-50.6)	31.7 (24.6-40.6)	−10.8 (−16.1 to −6.0)		47.9 (39.6-59.2)	46.6 (37.4-58.3)	−2.1 (−6.9 to 2.4)
EKFC_creatinine-cystatin_	42.5 (37.1-49.5)	31.0 (23.9-37.1)	−10.8 (−16.0 to −7.4)		46.6 (38.5-55.2)	44.4 (35.8-54.3)	−2.3 (−6.5 to 2.0)

Data are median (IQR).

CKD-EPI=Chronic Kidney Disease Epidemiology Collaboration estimating equation; EKFC=European Kidney Function Consortium estimating equation.

### Accuracy of GFR estimating equations when monitoring change in measured GFR over three years 

**Fig 1:**
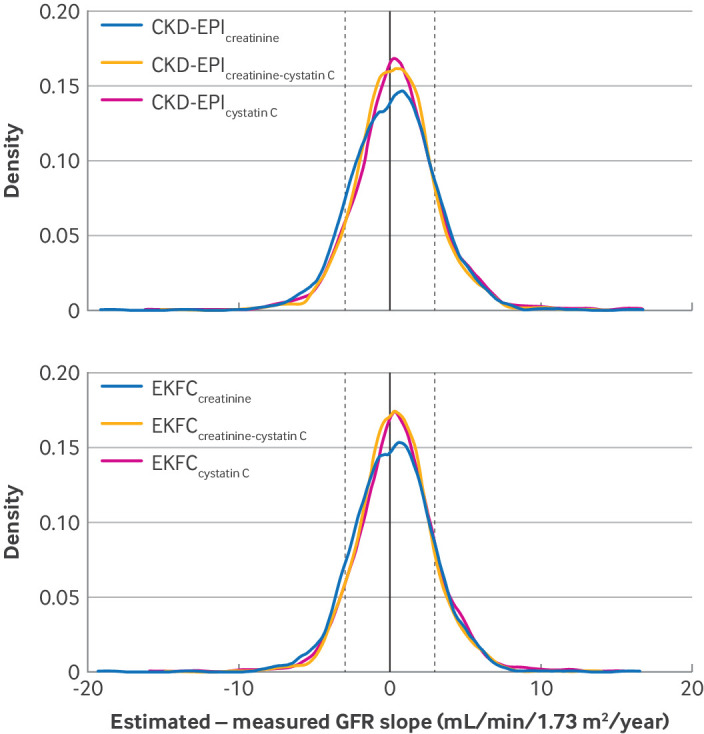
Distribution of error (estimated−measured glomerular filtration rate (GFR) slope) among study participants, with significant differences between slopes in measured and estimated GFR defined as ≥±3 mL/min/1.73 m^2^/year. Vertical line at zero indicates no difference in estimated and measured GFR slopes. Vertical dashed lines at +3 mL/min/1.73 m^2^/year and −3 mL/min/1.73 m^2^/year indicate the limits of acceptable agreement, with parts of the curve to the extreme left and right of these lines indicating the proportion of participants with a large error (supplementary file has examples of error calculations). Density represents 1/estimated−measured GFR slope. Top=data for the Chronic Kidney Disease Epidemiology Collaboration (CKD-EPI) estimating equations, with CKD-EPI_creatinine_, CKD-EPI_cystatin_, and CKD-EPI_creatinine-cystatin_ showing that 73.1%, 75.7%, and 78.6% of individuals, respectively, had change per year (slope) within ±3 mL/min/1.73 m^2^ of the slope change of measured GFR. Improving agreement is seen by an increase in the proportion of study participants within ±3 mL/min/1.73 m^2^/year. Bottom=data for European Kidney Function Consortium (EKFC) estimating equations, with EKFC_creatinine_, EKFC_cystatin_, and EKFC_creatinine-cystatin_ showing that 76.5%, 77.1%, and 80.2% of individuals, respectively, had change per year (slope) within ±3 mL/min/1.73 m^2^ of the slope change of measured GFR. The slight right shift of the curves in both figures is because estimated GFR more frequently underestimated than overestimated measured GFR change

The GFR estimating equations showed agreement, with change in measured GFR ranging from 72.6% to 80.2%. Agreement was defined as an estimated GFR slope within ±3 mL/min/1.73 m^2^/year of the slope of change for measured GFR (fig 1 and table 3).

**Table 3:** Performance of glomerular filtration rate (GFR) equations: number and percentage of participants with estimated GFR change per year (slope) within ±3 mL/min/1.73 m^2^ or within ±5% of slope change of measured GFR

Equation*	Difference in slope (estimated–measured GFR) within ±3 mL/min/1.73 m^2^			Difference in % change (estimated–measured GFR) within ±5%	
	No/total No	% (95% CI)		No/total No	% (95% CI)
CKD-EPI_creatinine_	640/875	73.1 (70.1 to 76.1)		553/875	63.2 (59.9 to 66.4)
CKD-EPI_cystatin_	662/875	75.7 (72.7 to 78.5)		573/875	65.5 (62.2 to 68.6)
CKD-EPI_creatinine-cystatin_	688/875	78.6 (75.8 to 81.3)		609/875	69.6 (66.4 to 72.6)
CKD-EPI(2021)_creatinine_	635/875	72.6 (69.5 to 75.5)		554/875	63.3 (60.0 to 66.5)
CKD-EPI(2021)_creatinine-cystatin_	683/875	78.1 (75.2 to 80.8)		608/875	69.5 (66.3 to 72.5)
EKFC_creatinine_	669/875	76.5 (73.5 to 79.2)		578/875	66.1 (62.8 to 69.2)
EKFC_cystatin_	675/875	77.1 (74.2 to 79.9)		618/875	70.6 (67.5 to 73.6)
EKFC_creatinine-cystatin_	702/875	80.2 (77.4 to 82.8)		641/875	73.3 (70.2 to 76.2)

* Estimating equations: CKD-EPI=Chronic Kidney Disease Epidemiology Collaboration; EKFC=European Kidney Function Consortium; CI=confidence interval.

The three combined equations, incorporating both creatinine and cystatin C, showed better performance at monitoring measured GFR over time than the CKD-EPI_creatinine_ equation (supplementary table 6). For example, the EKFC_creatinine-cystatin_ equation outperformed (P<0.001) the CKD-EPI_creatinine_ equation, with a difference of 7.1% (95% CI 4.5 to 9.7); that is, 7.1% more individuals had an estimated GFR slope within ±3 mL/min/1.73 m^2^/year of the measured GFR slope when using the EKFC_creatinine–cystatin_ equation compared with the CKD-EPI_creatinine_ equation. We found similar differences with the criterion of ≤±5% per year, although agreement was slightly lower (range 63.2% to 73.3%) than for the <±3 mL/min/1.73 m^2^/year analysis (table 3 and supplementary table 7). When stratified by sex, all equations showed agreement exceeding 72%, but the CKD-EPI_cystatin_ and EKFC_cystatin_ equations showed poorer performance (non-overlapping CIs) in women than in men (supplementary table 8).

The first sensitivity analysis (with up to seven estimated GFR measurements for each individual incorporated into a linear regression model) showed results similar to the original analysis, with slightly higher point estimates for the CKD-EPI_creatinine_ equation but overlapping CIs. In the second sensitivity analysis, with all estimated GFR values in the linear regression model for each person and additional measured GFR values (up to four measured GFR values for each individual in up to 215 participants) in a multilevel model, improved performance of the CKD-EPI equations, with a 7.4% improvement and non-overlapping CIs for CKD-EPI_creatinine_ compared with the original analysis (supplementary table 9).

### Accuracy of GFR estimating equations in detecting kidney disease progression over three years

Table 4 shows the performance of the GFR estimating equations in detecting a reduction in measured GFR of ≥25% together with a change in disease stage over three years. All equations had relatively low sensitivity (<54.1%) and high specificity (>90.4%) for detecting disease progression. For all equations, the negative predictive value (>90.0%) exceeded the positive predictive value (<61.2%).

**Table 4:** Performance of glomerular filtration rate (GFR) estimating equations in detecting a reduction in measured GFR ≥25% over three years and a reduction in disease category (eg, GFR category 3A to 3B)

Equation*	Reduction in measured GFR ≥25% over 3 years and change in disease category				Sensitivity (% (95% CI))	Specificity (% (95% CI))	Positive predictive value (% (95% CI))	Negative predictive value (% (95% CI))
	TP	FN	FP	TN				
CKD-EPI_creatinine_	75	64	63	673	54.0 (45.3 to 62.4)	91.4 (89.2 to 93.4)	54.3 (45.7 to 62.8)	91.3 (89.0 to 93.2)
CKD-EPI_cystatin_	71	68	68	668	51.1 (42.5 to 59.6)	90.8 (88.4 to 92.8)	51.1 (42.5 to 59.6)	90.8 (88.4 to 92.8)
CKD-EPI_creatinine-cystatin_	72	67	67	669	51.8 (43.2 to 60.3)	90.9 (88.6 to 92.9)	51.8 (43.2 to 60.3)	90.9 (88.6 to 92.9)
CKD-EPI(2021)_creatinine_	71	68	70	666	51.1 (42.5 to 59.6)	90.5 (88.1 to 92.5)	50.4 (41.8 to 58.9)	90.7 (88.4 to 92.7)
CKD-EPI(2021)_creatinine-cystatin_	75	64	65	671	54.0 (45.3 to 62.4)	91.2 (88.9 to 93.1)	53.6 (45.0 to 62.0)	91.3 (89.0 to 93.2)
EKFC_creatinine_	69	70	63	673	49.6 (41.1 to 58.2)	91.4 (89.2 to 93.4)	52.3 (43.4 to 61.0)	90.6 (88.2 to 92.6)
EKFC_cystatin_	63	76	51	685	45.3 (36.9 to 54.0)	93.1 (91.0 to 94.8)	55.3 (45.7 to 64.6)	90.0 (87.7 to 92.1)
EKFC_creatinine-cystatin_	69	70	44	692	49.6 (41.1 to 58.2)	94.0 (92.1 to 95.6)	61.1 (51.4 to 70.1)	90.8 (88.5 to 92.8)

* Estimating equations: CKD-EPI=Chronic Kidney Disease Epidemiology Collaboration; EKFC=European Kidney Function Consortium.

TP=true positive; FN=false negative; FP= false positive; TN=true negative; CI=confidence interval.

## Discussion

### Principal findings and comparison with other studies

We conducted a prospective, longitudinal study to examine how well contemporary GFR estimating equations, including those incorporating cystatin C, monitor change in measured GFR and detect disease progression over three years. For monitoring changes in measured GFR, all equations achieved >72.5% agreement with measured GFR, with limits of ≥±3 mL/min/1.73 m^2^/year (or >63.1% agreement with limits of >±5%/year) indicating large error, based on a previous study that considered that these errors would be clinically unacceptable.^4^ These data are in broad agreement with those reported by Padala et al who found changes in estimated GFR slope exceeding ±3 mL/min/1.73 m^2^/year from measured GFR slope in 15% of their cohort with the CKD-EPI_creatinine_ equation.^4^ The lower agreement for the >±5%/year change probably reflects the fact that this limit is slightly stricter than ≥+3 mL/ min/1.73 m^2^/year: at a median GFR of 48.1 mL/ min/1.73 m^2^, 5% equates to 2.4 mL/min/1.73 m^2^.

Equations incorporating only cystatin C performed at least equally for longitudinal monitoring as CKD-EPI_creatinine_ overall. Preliminary data suggested that the CKD-EPI_cystatin_ and EKFC_cystatin_ equations showed poorer performance in this respect in women. Poorer accuracy among women with GFR <60 mL/min/1.73 m^2^ was reported in the original cross sectional description of the EKFC_cystatin_ equation and confirmed more recently.^7^ ^11^ The explanation for this difference is unclear, and our study was not adequately powered to look at this question, which warrants further investigation. All equations with both creatinine and cystatin C, however, showed improved performance in monitoring measured GFR over time compared with equations based on one biomarker, with no differences in performance between men and women.

For the CKD-EPI equations, sensitivity analyses were undertaken with modelling approaches, as previously described, to include six monthly GFR estimates and additional measured GFR samples, where available.^4^ Although inclusion of additional estimated GFR measures in these sensitivity analyses had little impact, the modelling that included additional measured GFRs, where available, produced a substantial improvement in equation performance. This finding likely reflects improved precision of measured GFR assessment, which has its own intrinsic biological and analytical variability.^3^ Although these sensitivity analyses may more accurately reflect true change in estimated and measured GFR over time for the population through reduced measurement variability, these approaches may not reflect variability in individual sequential measurements that are likely to be seen in clinical practice in real time.

In this cohort, the median temporal change in estimated GFR was smaller than that for measured GFR. Others have also found that creatinine based equations underestimate the reduction in measured GFR.^14^ ^15^ ^16^ ^17^ ^18^ ^19^ For example, Xie et al^17^ found underestimation of the slope of iothalamate measured GFR reduction by 28%, with 42% of 542 individuals having an estimated GFR slope that differed from the measured GFR slope by ≥2 mL/min/1.73 m^2^/year. In a one year prospective study of 71 participants with autosomal dominant polycystic kidney disease, GFR estimating equations underestimated the reduction in iohexol measured GFR (mean change 8.4 mL/min/1.73 m^2^) by >50%.^18^ In a cross sectional analysis of the present cohort, a slope bias effect for GFR estimating equations was previously described, with positive bias (estimated–measured GFR) at lower levels of measured GFR (about <30-40 mL/min/1.73 m^2^) and negative bias at higher levels (about >40 mL/min/1.73 m^2^).^7^ In other words, when using equations, GFR, and therefore kidney function, is overestimated at lower levels of measured GFR and underestimated at higher levels of measured GFR. This effect was also reported for the CKD-EPI equations,^9^ and may partially explain the observed underestimation of measured GFR reduction by estimated GFR (ie, as GFR reduces over time, the intrinsic negative bias of the estimating equations relative to measured GFR reduces). This observation is further supported by the reduced negative bias of estimated GFR at follow-up in individuals who showed disease progression (table 2). A theoretical reason why creatinine based equations might underestimate the reduction in GFR is that as kidney disease progresses, increases in the proportion of creatinine excreted by renal tubules and extra-renal elimination of creatinine will rise, blunting the response of estimated GFR to declining kidney function.^20^ Creatinine is formed at a rate of 1.6-1.7% of creatine mass/day as a product of muscle creatine metabolism. Creatine is synthesised in a two part process; the first, synthesis of guanidinoacetate, occurs in the kidney. As renal functional mass decreases with disease progression, the production rate of guanidinoacetate (and ultimately creatinine) falls.^21^ Furthermore, as kidney disease progresses, muscle mass usually decreases, leading to a high prevalence of sarcopenia, itself driven in part by reduced synthesis of creatine. This process results in a change in the usual plasma creatinine-GFR relation.^22^ However, equations incorporating cystatin C also underestimated the reduction in GFR, despite plasma cystatin C concentrations being less dependent on muscle mass.

Disease progression (≥25% reduction in measured GFR in combination with a reduction in disease stage^2^) occurred in 15.9% of participants. All equations showed poor sensitivity (<54.1%) in detecting disease progression, with no clear differences between equations. This limitation potentially restricts the clinical ability to distinguish between stable patients and those with more progressive disease, and hence the ability to focus preventive interventions on higher risk individuals and timely referral for specialist care and renal replacement therapy. The relatively poor performance in detecting disease progression contrasts with the high concordance between estimated and measured GFR change when considering annual change within ±3 mL/min/1.73 m^2^/year (or ±5% per year). Several explanations are possible. As discussed above, all GFR estimating equations underestimated GFR reduction. Also, most participants did not show large reductions in GFR from baseline. When assessing the sensitivity and specificity of GFR estimating equations in detecting disease progression, results for both estimated and measured GFR were dichotomised to binary measures with no tolerance (ie, measured and estimated GFR either showed the difference of the magnitude investigated or did not). Sensitivity estimates were therefore based on a small number of individuals (15.9%) showing a larger reduction in measured GFR (≥25%) together with a change in disease stage who also showed this larger reduction in estimated GFR, rather than studying the whole group. Therefore, because of the continuous nature of GFR, binary measures of sensitivity would not fully capture the ability of GFR equations to detect a change in measured GFR in this cohort.

### Strengths and limitations of the study

The strengths of our research include the prospective design in a large, multicentre, adequately powered study and rigorous quality assured analyses of both reference and test measures in centralised laboratories. Multiple testing adjustment in the statistical analysis, such as Bonferroni correction, was not included. Given that estimated and measured GFR are independent and such corrections are conservative, this choice seems justified. By design, the study was limited to participants with an estimated GFR of 30-59 mL/min/1.73 m^2^ over three months before recruitment, but at baseline, 19% of those recruited had measured GFR ≥60 mL/min/1.73 m^2^. This variance is expected given the biological variation and performance characteristics, including a negative bias, of GFR estimating equations compared with measured GFR. Repeating these analyses, excluding those individuals with baseline measured GFR >60 mL/min/1.73 m^2^/year did not affect sensitivity and specificity estimates (data not shown). The point estimates of accuracy of all equations were reduced at follow-up. This finding likely reflects the fact that the P_30_ values were lower at lower GFR levels in this study population.^7^ Compared with other studies, the study population seemed to be fairly typical of a cohort with moderate chronic kidney disease in terms of the proportion of participants progressing to kidney failure^23^ and the rates of GFR reduction observed.^24^

Typical measures of diagnostic test accuracy, including sensitivity and specificity, were used to assess the ability of the equations to detect disease progression. As noted above, however, using binary test results has limitations, in particular because of the relatively small number of participants who showed disease progression (true positives): this finding should be considered when interpreting our results. Conversely, measures of agreement within ±3 mL/min/1.73 m^2^/year or ±5%/year will have been influenced by the majority of study participants that did not show a large change in measured GFR.

### Policy implications

Our findings have important implications for the use and interpretation of estimated GFR results in clinical care. Use of combined equations incorporating both creatinine and cystatin C significantly (P<0.001) improved the agreement between measured and estimated GFR over time. This finding supports the increasing body of cross sectional evidence suggesting that combined biomarker equations improve the accuracy of assessing GFR.^7^ ^9^ ^11^ ^12^ ^25^ Using combined equations would be a major change in clinical practice, but has the potential to substantially improve kidney care. As reported by others, however, in relation to creatinine based GFR estimates,^14^ ^15^ ^16^ ^17^ ^18^ ^19^ clinicians should be aware that estimates of GFR, including those based on cystatin C, underestimate the rate of measured GFR reduction. This concern is under-appreciated and clinically important. Plausible biological and methodological explanations for this underestimation exist. Future equation development work might look at the methodological components of this concern (eg, through different mathematical modelling). No evidence was found to suggest the superior performance of either the EKFC or CKD-EPI combined equations relative to the other in this study population with moderate chronic kidney disease.

### Conclusions

Estimating equations that included both creatinine and cystatin C showed better agreement with measured GFR than equations based on one biomarker when monitoring GFR over time. Use of these equations in clinical practice could improve monitoring of kidney function, with potential improvements in clinical care. Further research is needed to better understand the discrepancy between measured and estimated GFR change over time.

**What is already known on this topic**

Chronic kidney disease is monitored by glomerular filtration rate (GFR)
Measured GFR is not widely used whereas estimates of GFR based on levels of serum creatinine are commonly used but can be inaccurate
Cross sectional studies suggest that inclusion of cystatin C with creatinine in combined GFR estimating equations can improve the accuracy of assessing GFR

**What this study adds**

GFR estimating equations based on both cystatin C and creatinine showed better agreement with changes in measured GFR over three years than equations based on one biomarker
All GFR estimating equations underestimated the temporal reduction in GFR
Increased use of combined biomarker equations in clinical practice could improve disease monitoring and potentially clinical care

## Data Availability

An anonymised dataset and statistical code is available on the University of Birmingham eData Repository at https://doi.org/10.25500/edata.bham.00001497 (https://edata.bham.ac.uk/1497/).

## Web extra

Extra material supplied by authors

Web appendix: Supplementary file

## Notes

Contributors: KS was responsible for analysing data from the study and co-led writing and reviewing of the final report. AJSi was the study statistician and was involved in planning and developing the study, analysing the data, observing further analysis of the data, and writing and reviewing the final report. JB was co-principal investigator for recruitment in Leicester, provided critical review of the results throughout the study, reviewing and editing the study report. EAB was the Clinical Trials Unit co-investigator involved in planning and developing the study and responsible for oversight of study management. PC was involved in development of the study protocol, principal investigator for recruitment in Birmingham, critical review of the results throughout the study, and reviewing and editing the study report. RND was a co-principal investigator involved in all aspects of the research, from original proposal to submission of the final report, with primary responsibility for the iohexol measurements. JJD was the senior statistician and was involved in developing the study proposal, planning the analysis, overseeing the analysis, and contributed to the final report. GE was the lead research nurse for the study and was involved in protocol development, patient recruitment, data collection, and review of the final report. PAK was involved in development of the study protocol, principal investigator for recruitment in Salford, critical review of the results throughout the study, and reviewing and editing the study report. KK was co-principal investigator in Leicester and provided input in recruitment of diverse populations and reviewing the final report. FCL provided lay input, recommendations on patient involvement and patient representation on participation sheets, and study news. RO was the trial manager responsible for data quality, and day-to-day management and administrative support of the study. TP-H was one of the study statisticians and was involved in planning and developing the statistical methodology, analysing data, and writing and reviewing the final report. AP undertook laboratory analyses, data entry and analysis for the study, and contributed to writing and reviewing the final report. CR undertook laboratory analyses, statistical analysis, and contributed to writing and reviewing the final report. CCS was a principal investigator and contributed to the original proposal, patient recruitment in London, discussion of data analysis, and reviewing and editing the final report. BS took over as lead health economist from AJSu (AJSu left his post part way through the project). BS was involved in planning and developing the health economic statistical and economic analysis, supervising the economic evaluation systematic review update, and writing and reviewing the final report. AS undertook an economic evaluation systematic review update and the health economic statistical analysis, was involved in the economic analysis, and writing and reviewing the final report. PES was involved in the original proposal and development of the study protocol. PES was principal investigator for recruitment in Canterbury, critically reviewed results throughout the study, and reviewed and edited the final report. AJSu was the lead health economist in the early stages of the study. AJSu wrote the health economics section of the original research proposal and protocol, and was responsible for planning and conducting the initial economic evaluation systematic review. AJSu has reviewed the final report. MWT was a co-principal investigator and contributed to the grant application, protocol design, participant recruitment, and discussion of data interpretation as well as review of the final report. EJL was the chief investigator for the study and was involved in all aspects of the research from original proposal to submission of final report. EJL co-led writing of this paper. EJL is the study guarantor. EJL is the corresponding author for the study and attests that all listed authors meet the authorship criteria and that no others meeting the criteria have been omitted.

Transparency: The lead author (the manuscript’s guarantor) affirms that the manuscript is an honest, accurate, and transparent account of the study being reported; that no important aspects of the study have been omitted; and that any discrepancies from the study as originally planned (and, if relevant, registered) have been explained.

Dissemination to participants and related patient and public communities: Some of the results presented here have been published in *Health Technology Assessment*, the journal of the National Institute for Health and Care Research (Lamb et al. 6). The results have also been presented directly to clinicians and researchers at the 2025 national meeting of the United Kingdom Kidney Association (UKKA). Briefing papers describing the findings and their implications will be sent to Kidney Disease: Improving Global Outcomes, the National Institute of Health and Care Excellence, the Association for Laboratory Medicine, the Royal College of Pathologists, The Royal College of Physicians, Kidney Care UK, and UKKA.

Provenance and peer review: Not commissioned; externally peer reviewed.

## References

[1] National Institute for Health and Care Excellence. Chronic kidney disease: assessment and management [NG203]. https://www.nice.org.uk/guidance/ng203 34672500

[2] Kidney Disease Improving Global Outcomes. Clinical Practice Guideline for the Evaluation and Management of Chronic Kidney Disease. Kidney Int 2013;3:1-150. 10.1038/ki.2013.243 23989362

[3] Rowe C Sitch AJ Barratt J eGFR-C Study Group . Biological variation of measured and estimated glomerular filtration rate in patients with chronic kidney disease. Kidney Int 2019;96:429-35. 10.1016/j.kint.2019.02.021. 31084924

[4] Padala S Tighiouart H Inker LA . Accuracy of a GFR estimating equation over time in people with a wide range of kidney function. Am J Kidney Dis 2012;60:217-24. 10.1053/j.ajkd.2012.01.024 22495467 PMC3399947

[5] Lamb EJ Brettell EA Cockwell P eGFR-C study group . The eGFR-C study: accuracy of glomerular filtration rate (GFR) estimation using creatinine and cystatin C and albuminuria for monitoring disease progression in patients with stage 3 chronic kidney disease--prospective longitudinal study in a multiethnic population. BMC Nephrol 2014;15:13. 10.1186/1471-2369-15-13 24423077 PMC3898236

[6] Lamb EJ Barratt J Brettell EA . Accuracy of glomerular filtration rate estimation using creatinine and cystatin C for identifying and monitoring moderate chronic kidney disease: the eGFR-C study. Health Technol Assess 2024;28:1-169. 10.3310/HYHN1078. 39056437 PMC11331378

[7] Lamb EJ Barratt J Brettell EA . Test accuracy of glomerular filtration rate estimation with creatinine and cystatin C in adults with moderate chronic kidney disease: prospective cohort study. BMJ Med 2026;5:e001827. 10.1136/bmjmed-2025-001827. 41586348 PMC12829397

[8] Levey AS Stevens LA Schmid CH CKD-EPI (Chronic Kidney Disease Epidemiology Collaboration) . A new equation to estimate glomerular filtration rate. Ann Intern Med 2009;150:604-12. 10.7326/0003-4819-150-9-200905050-00006 19414839 PMC2763564

[9] Inker LA Schmid CH Tighiouart H CKD-EPI Investigators . Estimating glomerular filtration rate from serum creatinine and cystatin C. N Engl J Med 2012;367:20-9. 10.1056/NEJMoa1114248. 22762315 PMC4398023

[10] Pottel H Björk J Courbebaisse M . Development and Validation of a Modified Full Age Spectrum Creatinine-Based Equation to Estimate Glomerular Filtration Rate : A Cross-sectional Analysis of Pooled Data. Ann Intern Med 2021;174:183-91. 10.7326/M20-4366. 33166224

[11] Pottel H Björk J Rule AD . Cystatin C-Based Equation to Estimate GFR without the Inclusion of Race and Sex. N Engl J Med 2023;388:333-43. 10.1056/NEJMoa2203769. 36720134

[12] Inker LA Eneanya ND Coresh J Chronic Kidney Disease Epidemiology Collaboration . New Creatinine- and Cystatin C-Based Equations to Estimate GFR without Race. N Engl J Med 2021;385:1737-49. 10.1056/NEJMoa2102953. 34554658 PMC8822996

[13] National Kidney Foundation . K/DOQI clinical practice guidelines for chronic kidney disease: evaluation, classification, and stratification. Am J Kidney Dis 2002;39(Suppl 1):S1-266. 11904577

[14] Lewis J Greene T Appel L AASK Study Group . A comparison of iothalamate-GFR and serum creatinine-based outcomes: acceleration in the rate of GFR decline in the African American Study of Kidney Disease and Hypertension. J Am Soc Nephrol 2004;15:3175-83. 10.1097/01.ASN.0000146688.74084.A3. 15579521

[15] Fontseré N Salinas I Bonal J . Are prediction equations for glomerular filtration rate useful for the long-term monitoring of type 2 diabetic patients? Nephrol Dial Transplant 2006;21:2152-8. 10.1093/ndt/gfl221 16702203

[16] Rossing P Rossing K Gaede P Pedersen O Parving HH . Monitoring kidney function in type 2 diabetic patients with incipient and overt diabetic nephropathy. Diabetes Care 2006;29:1024-30. 10.2337/dc05-2201 16644632

[17] Xie D Joffe MM Brunelli SM . A comparison of change in measured and estimated glomerular filtration rate in patients with nondiabetic kidney disease. Clin J Am Soc Nephrol 2008;3:1332-8. 10.2215/CJN.05631207 18667734 PMC2518808

[18] Ruggenenti P Gaspari F Cannata A GFR-ADPKD Study Group . Measuring and estimating GFR and treatment effect in ADPKD patients: results and implications of a longitudinal cohort study. PLoS One 2012;7:e32533. 10.1371/journal.pone.0032533. 22393413 PMC3291245

[19] Tent H Waanders F Krikken JA . Performance of MDRD study and CKD-EPI equations for long-term follow-up of nondiabetic patients with chronic kidney disease. Nephrol Dial Transplant 2012;27(Suppl 3):iii89-95. 10.1093/ndt/gfr235. 21562145

[20] Shemesh O Golbetz H Kriss JP Myers BD . Limitations of creatinine as a filtration marker in glomerulopathic patients. Kidney Int 1985;28:830-8. 10.1038/ki.1985.205 2418254

[21] Post A Tsikas D Bakker SJL . Creatine is a Conditionally Essential Nutrient in Chronic Kidney Disease: A Hypothesis and Narrative Literature Review. Nutrients 2019;11:1044. 10.3390/nu11051044. 31083291 PMC6567063

[22] Sabatino A Cuppari L Stenvinkel P Lindholm B Avesani CM . Sarcopenia in chronic kidney disease: what have we learned so far? J Nephrol 2021;34:1347-72. 10.1007/s40620-020-00840-y. 32876940 PMC8357704

[23] Keith DS Nichols GA Gullion CM Brown JB Smith DH . Longitudinal follow-up and outcomes among a population with chronic kidney disease in a large managed care organization. Arch Intern Med 2004;164:659-63. 10.1001/archinte.164.6.659 15037495

[24] Hemmelgarn BR Zhang J Manns BJ . Progression of kidney dysfunction in the community-dwelling elderly. Kidney Int 2006;69:2155-61. 10.1038/sj.ki.5000270. 16531986

[25] Fu EL Levey AS Coresh J . Accuracy of GFR estimating equations based on creatinine, cystatin C or both in routine care. Nephrol Dial Transplant 2024;39:694-706. 10.1093/ndt/gfad219. 37813817

