# Supplementary material for: Accuracy of glomerular filtration rate estimation based on creatinine and cystatin C for monitoring moderate chronic kidney disease in adults: prospective, longitudinal cohort study
Source: BMJ. 2026 Mar 19;392:e085005. doi: 10.1136/bmj-2025-085005 (PMC13000885; doi:10.1136/bmj-2025-085005)
Supplement: Supplementary file 1 — Web appendix: Supplementary file [file scak085005.ww.pdf]

Supplementary file for Scandrett et al, Accuracy of glomerular filtration rate estimation using creatinine and cystatin C for monitoring moderate chronic kidney disease in adults: prospective, longitudinal, cohort study

Recruitment, inclusion and exclusion criteria

The aim of the study was to recruit 1300 participants in total. In total 29,845 people were screened for potential suitability for study inclusion: 15,340 were deemed unsuitable at an early stage from informatics and clinic lists due to not having chronic kidney disease (CKD) stage 3. Exclusions were a history of untoward reaction to iodinated contrast media or allergy to topical iodine, pregnant or breast-feeding, known current alcohol or drug abuse, kidney transplant recipient, people whose life expectancy would make study completion unlikely,[1] inability to consent e.g. due to cognitive impairment, inability to comply with study schedule and follow-up, amputation of whole or part limb, recent (last 6 months) episode of acute kidney injury,[2] and sickle cell disease. People considered suitable for study inclusion (n=6,209) were approached in person and/or sent participant information sheets. A further 4,000 individuals were contacted through their primary care provider. Reasons for declining to participate were recorded in 928 cases. The major reasons for declining included that they were not interested in research; that they had too many medical appointments; that the five hour appointment time was too long; that too much travel was involved; that they were already in other research studies; and that the study involved too many injections. A total of 1,229 participants were recruited to the study between April 2014 and January 2017. Recruitment was primarily from secondary/tertiary care, with 72 patients being recruited from primary care.

Adults with stage 3 CKD were recruited to the study at six centres in England (Birmingham, Canterbury, Derby, Leicester, Salford and London [Kings College Hospital]). Individuals aged 18 years and older having stage 3 CKD (estimated glomerular filtration rates [GFR] between 30 and 59 mL/min/1.73 m^2 inclusive, obtained using the Modification of Diet in Renal Disease (MDRD) Study equation,[3] sustained over at least 3 months prior to recruitment) were included. The MDRD equation was used as this was standard practice in England at the time of study commencement.

Sampling and data collection

Participants were asked to attend hospital in the morning having been advised to consume a light breakfast (no meat or fish). A clinical and drug history was recorded using a standardised questionnaire taken by research nurses on the day of hospital attendance. Vascular disease was defined as the presence of myocardial infarction (MI, including ST-elevation myocardial infarction, [STEMI] and non-ST elevation myocardial infarction [NSTEMI]), angina, congestive cardiac failure (heart failure) or a requirement for coronary intervention (angioplasty, coronary artery bypass graft or pacemaker), cerebrovascular or peripheral vascular disease. Information on ethnicity was gathered using a modified version of the 2011 UK Census Questionnaire, with ethnicity being mapped to the following codes: Caucasian 31, 32, 33, 34; South-Asian 39, 40, 41; Black 44, 45, 46. Height was measured to the nearest 0.1 cm with a rigid stadiometer. Body weight was measured in light indoor clothing to the nearest 0.1 kg. Brachial blood pressure was measured as recommended by the British and Irish Hypertension Society (https://bihs.org.uk/, accessed 24th June 2025) three times in the sitting position using standardised Omron M7 digital sphygmomanometers (Omron Healthcare, Milton Keynes, UK). The average of the second and third blood pressure readings was recorded.

Baseline blood was taken for serum creatinine and cystatin C, and a urine sample was collected for albumin to creatinine ratio (ACR). Blood samples were collected using standard venepuncture and phlebotomy procedures including the use of a tourniquet. Blood was collected in appropriate Greiner Vacuette™ tubes (www.gbo.com, accessed 24th June 2025) following the manufacturer's recommended order of draw. The urine sample was taken into a plain sterilin pot. Samples were transported to the local laboratory, where plasma/serum was separated within 4-6 h of venepuncture by centrifuging at 2000 g for 10 mins. Aliquots of serum/plasma and urine were then stored at -80°C pending transportation to the central laboratories (St. Thomas's Hospital, London [iohexol, isotope-dilution mass spectrometry (ID-MS) creatinine] or Canterbury [enzymatic creatinine, cystatin C, albumin to creatinine ratio (ACR)] depending on analyte) and analysis.

Prior to analysis, samples were thawed at room temperature, mixed by inversion and centrifuged. Each of the biomarker analyses was undertaken by a single operator blinded to participant data using a single instrument. Creatinine and cystatin C measurements were undertaken in an accredited laboratory by scientists registered with the Health and Care Professions Council and blinded to the results of the reference test. Scientists undertaking the reference test were blinded to the results of the index tests.

GFR was measured using an iohexol clearance method.[4] A 5 mL bolus of Omnipaque 240 (518 g/L iohexol corresponding to 240 g/L of iodine, GE Healthcare (https://www.gehealthcare.co.uk/, accessed 24th June 2025) followed by 10 mL of normal saline was injected into the antecubital vein. Blood samples were collected at 5, 120, 180 and 240 minutes after injection. Exact time of the samples in relation to the bolus injection was accurately recorded. Participants were allowed free access to fluids during the collection procedure but were asked to refrain from protein intake (i.e. biscuits/toast would be permitted) and to refrain from excessive exercise. Samples were stored at -80°C prior to analysis. Iohexol was determined using an ID-MS method[4] and GFR calculated.[5]

GFR was estimated using serum creatinine and/or cystatin C results measured on the baseline blood sample obtained during the iohexol procedure. GFR was estimated using the following equations: the Chronic Kidney Disease Epidemiology Collaboration ($CKD\text{-}EPI_{creatinine}$) equation,[6] the CKD-EPI cystatin C-containing equations ($CKD\text{-}EPI_{cystatin}$, $CKD\text{-}EPI_{creatinine\text{-}cystatin}$),[7] the European Kidney Function Consortium ($EKFC_{creatinine}$ $EKFC_{cystatin,}$ $EKFC_{creatinine\text{-}cystatin}$) equations,[8, 9] and the 2021 revisions of the CKD-EPI equations ($CKD\text{-}EPI(2021)_{creatinine}$ and $CKD\text{-}EPI(2021)_{creatinine\text{-}cystatin}$).[10] Creatinine was measured by an enzymatic method and cystatin C by a turbidimetric immunoassay (both Abbott Diagnostics Ltd, https://www.abbott.co.uk/, accessed 24th June 2025).[11, 12]

Participants were followed for 3 years, with all measurements being repeated at 36 months. All participants also had GFR estimated using all of the above equations at 6 monthly intervals (7 estimated GFRs in total). Some participants underwent additional measured GFR testing at one and two years (4 measured GFRs in total)(see Supplementary Figure 1).

<u>Examples of estimated versus measured GFR slope error calculations</u>

The outcome of interest was the difference between the measured and estimated GFR slope calculated by subtracting the observed measured GFR slope from the observed estimated GFR slope. For an estimated GFR equation that accurately tracks measured GFR, the difference (error) between slopes should be small (Supplementary equation 1), defined in this study as <3 mL/min/1.73 m^2.

Supplementary equation 1.

$$Error\ value = estimated\ GFR\ slope - measured\ GFR\ slope$$

For example, patient A has estimated GFR 60 at baseline and measured GFR 65 at baseline. At 3-year follow-up, their estimated GFR is 50 and their measured GFR is 52. The estimated GFR and measured GFR slopes (mL/min/1.73 m^2) for this patient are:

$$measured\ GFR\ slope = \frac{measured\ GFR\ at\ follow\ up - measured\ GFR\ at\ baseline}{Years\ between\ baseline\ and\ follow\ up} = \frac{52 - 65}{3} = -4.3$$

$$estimated\ GFR\ slope = \frac{estimated\ GFR\ at\ follow\ up - estimated\ GFR\ at\ baseline}{Years\ between\ baseline\ and\ follow\ up} = \frac{50 - 60}{3} = -3.3$$

Using these slope values, we can calculate the error value:

$$Error\ value = -3.3 - (-4.3) = 1$$

Therefore, this patient's error value is not large, using the criteria of a difference between estimated GFR and measured GFR of ≥3 mL/minute/1.73 m^2 or ≤-3 mL/minute/1.73 m^2 per year to signify a 'large error'.

Patient B has estimated GFR 60 at baseline and measured GFR 65 at baseline. At 3-year follow-up their estimated GFR is 50 and their measured GFR is 66. The estimated GFR and measured GFR slopes (mL/min/1.73 m^2) for this patient are:

$$measured\ GFR\ slope = \frac{measured\ GFR\ at\ follow\ up - measured\ GFR\ at\ baseline}{Years\ between\ baseline\ and\ follow\ up} = \frac{66 - 65}{3} = 0.3$$

$$estimated\ GFR\ slope = \frac{estimated\ GFR\ at\ follow\ up - estimated\ GFR\ at\ baseline}{Years\ between\ baseline\ and\ follow\ up} = \frac{50 - 60}{3} = -3.3$$

Using these slope values, we can calculate the error value:

$$Error\ value = -3.3 - 0.3 = -3.6$$

Therefore, this patient's error value is large, using the criteria of a difference between estimated GFR and measured GFR of ≥3 mL/minute/1.73 m^2 or ≤-3 mL/minute/1.73 m^2 per year to signify a 'large error'.

Supplementary Figure 1. Consolidated Standards of Reporting Trials (Consort) flow diagram illustrating recruitment and follow-up in the study.

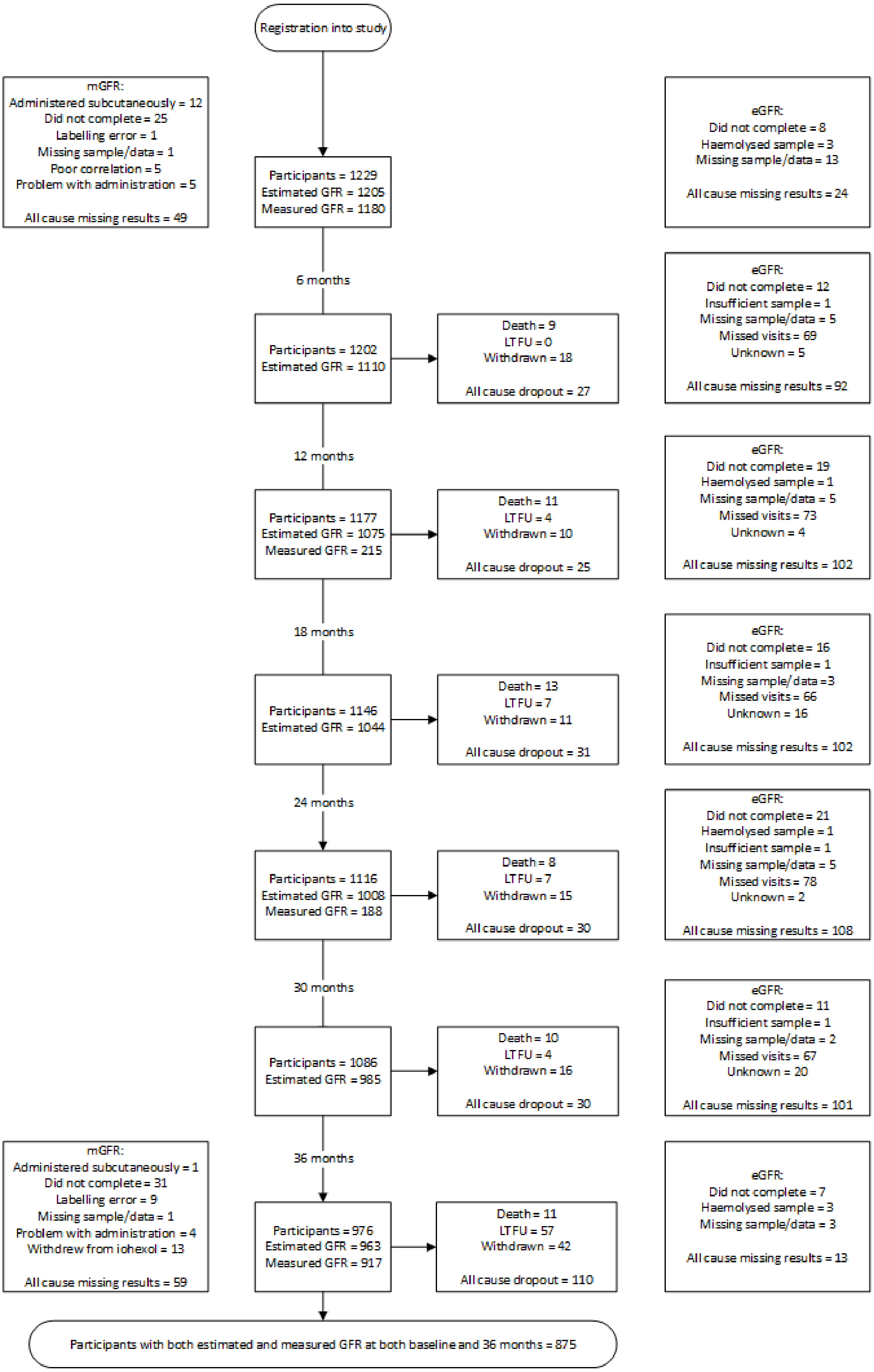

Supplementary Table 1. Equations used to estimate glomerular filtration rate (GFR). Age is given in years, serum creatinine (SCr) in μmol/L, serum cystatin C (SCys) in mg/L, weight in kilograms.

Abbreviation	GFR equation
CKD-EPI$_{creatinine}$[6]	GFR (mL/min/1.73 m^2) = 141 x min(SCr x 0.01131/κ, 1)$^{\alpha}$ x max(SCr x 0.01131/κ, 1)$^{-1.209}$ x 0.993^{age} x 1.018 [if female] x 1.159 [if black] , where SCr is serum creatinine, κ is 0.7 for females and 0.9 for males, α is -0.329 for females and -0.411 for males, min indicates the minimum of SCr/κ or 1, and max indicates the maximum of SCr/κ or 1
CKD-EPI$_{cystatin}$[7]	GFR (mL/min/1.73 m^2) = 133 x min(SCys/0.8, 1)$^{-0.499}$ x max(SCys/0.8, 1)$^{-1.328}$ x 0.996^{Age} x 0.932 [if female], where min indicates the minimum of SCys/κ or 1, and max indicates the maximum of SCys/κ or 1.
CKD-EPI$_{creatinine-cystatin}$[7]	GFR (mL/min/1.73 m^2) = 135 x min(SCr x 0.01131/κ, 1)$^{\alpha}$ x max(SCr x 0.01131/κ, 1)$^{-0.601}$ x min(SCys/0.8, 1)$^{-0.375}$ x max(SCys/0.8, 1)$^{-0.711}$ x 0.995^{Age} x 0.969 [if female] x 1.08 [if black], where SCr is serum creatinine, SCys is serum cystatin C, κ is 0.7 for females and 0.9 for males, α is -0.248 for females and -0.207 for males, min indicates the minimum of SCr/κ or 1, and max indicates the maximum of SCr/κ or 1.
CKD-EPI(2021)$_{creatinine}$[10]	GFR (mL/min/1.73 m^2) = 142 x min(Scr/k,1)$^{\alpha}$ x max(Scr/k,1)$^{-1.200}$ x 0.9938^{age} x 1.012 [if female], where Scr is serum creatinine, k is 0.7 for females and 0.9 males, α is -0.241 for females and -0.302 for males, min indicates the minimum of Scr/k or 1, max indicates the maximum of Scr/k or 1
CKD-EPI(2021)$_{creatinine-cystatin}$[10]	GFR (mL/min/1.73 m^2) = 135 x min(Scr/k,1)$^{\alpha}$ x max(Scr/k,1)$^{-0.544}$ x min(Scys/0.8,1)$^{-0.323}$ x max(Scys/0.8,1)$^{-0.778}$ x 0.9961^{age} x 0.963 [if female], where Scr is serum creatinine Scys is serum cystatin C, k is 0.7 for females and 0.9 males, α is -0.219 for females and -0.144 for males, min indicates the minimum of Scr/k or 1, max indicates the maximum of Scr/k or 1
EKFC$_{creatinine}$[8]	GFR (mL/min/1.73 m^2) = 107.3 × (SCr/Q)$^{-0.322}$ for age 2 to 40 and SCr/Q <1<br>GFR (mL/min/1.73 m^2) = 107.3 × (SCr/Q)$^{-1.132}$ for age 2 to 40 and SCr/Q $\geq$1

Abbreviation	GFR equation
	GFR (mL/min/1.73 m^2) = 107.3 x $(SCr/Q)^{-0.322}$ × $0.990^{(Age-40)}$ for age >40 and SCr/Q <1
	GFR (mL/min/1.73 m^2) = 107.3 × $(SCr/Q)^{-1.132}$ × $0.990^{(Age-40)}$ for age >40 and SCr/Q $\geq$1
	where Q values are the mean or median serum creatinine concentration for age/gender-specific healthy reference populations
$EKFC_{cystatin}$[9]	GFR (mL/min/1.73 m^2) = 107.3 × $(SCys/0.83)^{-0.322}$ for age 18 to 40 and SCys/0.83 < 1
	GFR (mL/min/1.73 m^2) = 107.3 × $(SCys/0.83)^{-1.132}$ for age 18 to 40 and SCys/0.83 $\geq$ 1
	GFR (mL/min/1.73 m^2) = 107.3 × $(SCys/0.83)^{-0.322}$ × $0.990^{(Age-40)}$ for age > 40, to age 50, and SCys/0.83 < 1
	GFR (mL/min/1.73 m^2) = 107.3 × $(SCys/0.83)^{-1.132}$ × $0.990^{(Age-40)}$ for age > 40, to age 50, and SCys/0.83 $\geq$ 1
	GFR (mL/min/1.73 m^2) = 107.3 × $(SCys/Q)^{-0.322}$ × $0.990^{(Age-40)}$ for age > 50 and SCys/Q < 1
	GFR (mL/min/1.73 m^2) = 107.3 × $(SCys/Q)^{-1.132}$ × $0.990^{(Age-40)}$ for age > 50 and SCys/Q $\geq$ 1
	where Q = 0.83 + 0.005 × (Age - 50)
$EKFC_{creatinine\text{-}cystatin}$[9]	GFR (mL/min/1.73 m^2) = ($EKFC_{creatinine}$ + $EKFC_{cystatin}$)/2

Supplementary Table 2. 2x2 table depicting true positive, false negative, false positive and true negative classifications for calculation of test accuracy metrics to determine which GFR-estimating equation (eGFR) most accurately detects change in measured GFR (mGFR) and disease progression.

	≥25% decline in mGFR + decline in mGFR disease category	<25% decline in mGFR or no decline in mGFR disease category
≥25% decline in eGFR + decline in eGFR disease category	True positive	False positive
<25% decline in eGFR or no decline in eGFR disease category	False negative	True negative

Supplementary Table 3. Study entry characteristics of participants with baseline data (n = 1167) and those included in the monitoring study (n = 875).

Characteristics	All participants with mGFR and eGFR at baseline*	All participants with mGFR and eGFR at baseline and 3 year follow up**
n	1,167	875
Age, y	67.5 [58.3, 74.5]	67.1 [58.1, 73.6]
M:F, n	680:487	505:370
Ethnicity		
Caucasian, n (%)	1,014 (86.9)	773 (88.3)
African-Caribbean, n (%)	60 (5.1)	36 (4.1)
South Asian, n (%)	66 (5.7)	46 (5.3)
Other, n (%)***	27 (2.3)	20 (2.3)
Height, cm	170 [162, 176]	170 [163, 176]
Weight, kg	84.1 [72.5, 97.3]	84.7 [73, 97.2]
Du Bois body surface area, m^2	1.96 [1.80, 2.10]	1.96 [1.81, 2.11]
Body mass index, kg/m^2	29.0 [25.8, 33.3]	29.0 [25.7, 33.4]
Medication recorded (n, %)	thiazide diuretic (123, 10.5), loop diuretic (180, 15.4), potassium sparing diuretic (26, 2.2), beta-blocker (314, 26.9), CCB (376, 30.6), ACE inhibitor (411, 35.2), ARB (348, 29.8), alpha-blocker (153, 13.1), HMG CoA reductase inhibitor (635, 54.4), allopurinol (137, 11.7), antiplatelet drugs (367, 31.4)	thiazide diuretic (94, 10.7), loop diuretic (114, 13.0), potassium sparing diuretic (18, 2.1), beta-blocker (223, 25.5), CCB (273, 31.2), ACE inhibitor (317, 36.2), ARB (267, 30.5), alpha-blocker (111, 12.7), HMG CoA reductase inhibitor (463, 52.9), allopurinol (105, 12.0),

Characteristics	All participants with mGFR and eGFR at baseline*	All participants with mGFR and eGFR at baseline and 3 year follow up**
		antiplatelet drugs (258, 29.5)
Comorbidity recorded (n, %)****	diabetes mellitus (324, 27.8), ischaemic heart disease (177, 15.2), angina (88, 7.5), heart failure (55, 4.7), cerebrovascular disease (85, 7.3), TIA (48, 4.1), stroke (37, 3.2), HBV (18, 1.5), malignancy (191, 16.4)	diabetes mellitus (220, 25.1), ischaemic heart disease (120, 13.7), angina (60, 6.9), heart failure (30, 3.4), cerebrovascular disease (56, 6.4), TIA (29, 3.3), stroke (26, 3.0), HBV (14, 1.6), malignancy (134, 15.3)
Smoking status		
Non-smoker, n (%)	590 (50.6)	453 (51.8)
Current smoker, n (%)	101 (8.7)	63 (7.2)
Former smoker, n (%)	474 (40.6)	357 (40.8)
Unknown, n (%)	2 (0.2)	2 (0.2)
Urine albumin concentration		
<3 mg/mmol, n (%)	483 (41.4)	375 (42.9)
3-30 mg/mmol, n (%)	396 (33.9)	295 (33.7)
>30 mg/mmol, n (%)	269 (23.1)	195 (22.3)
Missing, n (%)	19 (1.6)	10 (1.1)
Serum creatinine, µmol/L	129 [107, 154] n=1167	132 [109, 162] n=875
Serum cystatin C, mg/L (Siemens)	1.53 [1.28, 1.81] n=1167	1.55 [1.26, 1.84] n=875
CKD GFR category (‘stage’) at baseline based on measured GFR, n (%)		
1 n (%)	7 (0.6)	5 (0.6)
2, n (%)	204 (17.5)	163 (18.6)
3A, n (%)	452 (38.7)	366 (41.8)

Characteristics	All participants with mGFR and eGFR at baseline*	All participants with mGFR and eGFR at baseline and 3 year follow up**
3B, n (%)	434 (37.2)	303 (34.6)
4, n (%)	68 (5.8)	38 (4.3)
5, n (%)	2 (0.2)	0 (0)
Measured GFR, mL/min/1.73 m^2	47.0 [38.7, 56.4]	48.1 [40.2, 57.2]

Abbreviations: ACE, angiotensin converting enzyme; ARB, angiotensin-2 receptor blocker; CCB, calcium channel blocker; CKD, chronic kidney disease; GFR, glomerular filtration rate; HMG CoA, hydroxymethyl glutaryl CoA reductase; TIA, transient ischaemic attack; HBV, hepatitis B virus.

Values for continuous data are shown as median [IQR].

*Participants with mGFR and ANY eGFR result at baseline were included
** Participants with mGFR and ANY eGFR result at both baseline and follow-up were included
***Other includes participants with ethnic background other than Caucasian, South-Asian or African-Caribbean, plus three individuals where data was not recorded.
****only comorbidities affecting a minimum of 20 individuals in the baseline recruited cohort are listed

Supplementary Table 4. Performance of GFR estimating equations compared to measured GFR at baseline and 3 years follow-up.

Equation	Median (IQR) GFR, mL/min/1.73 m^2 at baseline (n=875)	Median (IQR) GFR, mL/min/1.73 m^2 at follow-up (n=875)	Bias (estimated minus measured GFR), median difference (IQR), mL/min/1.73 m^2 at baseline (n=875)	Bias (estimated minus measured GFR), median difference (IQR), mL/min/1.73 m^2 at follow-up (n=875)	Accuracy, percentage of estimates within 30% of measured GFR (P_{30}) (95% CI) at baseline (n=875)	Accuracy, percentage of estimates within 30% of measured GFR (P_{30}) (95% CI) at follow-up (n=875)
Measured GFR	48.1 (40.2, 57.2)	43.6 (35.0, 53.3)	na	na	na	na
$CKD\text{-}EPI_{creatinine}$	45.7 (37.7, 54.2)	42.0 (32.2, 52.1)	-3.3 (-8.5, 3.3)	-2.6 (-7.6, 2.9)	89.8 (87.6, 91.8)	86.1 (83.6, 88.3)
$CKD\text{-}EPI_{cystatin}$	43.6 (35.0, 54.3)	40.4 (30.2, 52.3)	-3.6 (-9.2, 1.6)	-2.3 (-8.0, 2.7)	91.0 (88.9, 92.8)	84.9 (82.4, 87.2)
$CKD\text{-}EPI_{creatinine\text{-}cystatin}$	43.4 (35.9, 53.3)	40.6 (31.0, 51.4)	-4.0 (-8.5, 1.1)	-3.2 (-7.4, 1.6)	95.0 (93.3, 96.3)	89.9 (87.8, 91.9)

CKD-EPI(2021)$_{creatinine}$	48.3<br>(39.5, 57.2)	44.6<br>(34.3, 55.2)	-0.9<br>(-6.3, 6.0)	-0.3<br>(-5.5, 5.6)	88.7<br>(86.4, 90.7)	85.7<br>(83.2, 88.0)
CKD-EPI(2021)$_{creatinine-cystatin}$	46.1<br>(37.8, 56.6)	43.1<br>(33.0, 54.7)	-1.3<br>(-6.1, 3.7)	-0.4<br>(-5.3, 4.4)	95.4<br>(93.8, 96.7)	90.6<br>(88.5, 92.5)
EKFC$_{creatinine}$	43.8<br>(36.5, 51.5)	40.4<br>(31.6, 49.0)	-4.9<br>(-10.4, 1.1)	-4.1<br>(-9.3, 1.0)	89.5<br>(87.3, 91.4)	88.1<br>(85.8, 90.2)
EKFC$_{cystatin}$	47.2<br>(39.1, 57.8)	44.0<br>(34.5, 56.0)	-0.1<br>(-5.6, 5.4)	1.4<br>(-4.4, 6.3)	90.9<br>(88.8, 92.7)	87.4<br>(85.0, 89.6)
EKFC$_{creatinine-cystatin}$	45.6<br>(38.2, 54.3)	42.3<br>(33.1, 51.9)	-2.2<br>(-7.0, 2.4)	-1.3<br>(-5.8, 2.9)	94.9<br>(93.2, 96.2)	93.0<br>(91.1, 94.6)

na, not applicable

Supplementary Table 5. Study entry characteristics of participants categorised by those that did and did not show kidney disease progression during the study.

Characteristics		Non-progressors, n=736	Progressors, n=139
Age, y		67.4 [58.4, 73.5]	66.2 [55.9, 74.4]
Male:Female, n		412:324	93:46
Ethnicity, n (%)	White	650 (88.3)	123 (88.5)
	Black	33 (4.5)	3 (2.2)
	South Asian	37 (5.0)	9 (6.5)
	Other*	16 (2.2)	4 (2.9)
Height, cm		170 [162, 176]	170 [164, 178]
Weight, kg		84.1 [72.4, 97.1]	86.7 [76.6, 97.3]
Body mass index, kg/m^2		28.9 [25.6, 33.2]	29.5 [26.3, 34.0]
Urine albumin concentration, n (%)	<3 mg/mmol	347 (47.2)	28 (20.1)
	3-30 mg/mmol	244 (33.2)	57 (26.7)
	>30 mg/mmol	138 (18.8)	57 (41.0)
	Missing	7 (1.0)	3 (2.2)
Measured GFR, mL/min/1.73 m^2		48.4 [40.4, 57.8]	47.7 [39.1, 55.1]

Characteristics		Non-progressors, n=736	Progressors, n=139
Serum creatinine, µmol/L		126.0 [104.5, 150.0]	137.0 [120.0, 161.0]
Serum cystatin C, mg/L		1.47 [1.24, 1.72]	1.61 [1.41, 1.92]
CKD GFR category (‘stage’) at baseline based on measured GFR, n (%)	1	4 (0.5)	1 (0.7)
	2	147 (20.0)	16 (11.5)
	3A	296 (40.2)	70 (50.4)
	3B	252 (34.2)	51 (36.7)
	4	37 (5.0)	1 (0.7)
	5	0 (0.0)	0 (0.0)
Diabetes, n (%)		171 (23.2)	49 (35.3)
Smoking, n (%)	Non-smoker	377 (51.2)	76 (54.7)
	Current smoker	55 (7.5)	8 (5.8)
	Former smoker	302 (41.0)	55 (39.6)
	Unknown	2 (0.3)	0 (0.0)

Values for continuous data are shown as median [IQR].

*Other includes participants with ethnic background other than Caucasian, South-Asian or African-Caribbean, plus three individuals where data was not recorded

Supplementary Table 6. Comparison of each GFR estimating equation's absolute performance (rows, test B) over time compared to each of the CKD-EPI equations (columns, test A).

**Test B**	**Test A**		
	CKD-EPI$_{creatinine}$	CKD-EPI$_{cystatin}$	CKD-EPI$_{creatinine\text{-}cystatin}$
CKD-EPI$_{creatinine}$			
CKD-EPI$_{cystatin}$	2.5 (-0.9, 5.9) p=0.16		
CKD-EPI$_{creatinine\text{-}cystatin}$	**5.5 (2.8, 8.2) p<0.001**	**3.0 (0.7, 5.3) p=0.010**	
CKD-EPI(2021)$_{creatinine}$	-0.6 (-1.6, 0.5) p=0.33	-3.1 (-6.6, 0.4) p=0.08	-6.1 (-8.8, -3.3) p<0.001
CKD-EPI(2021)$_{creatinine\text{-}cystatin}$	**4.9 (2.1, 7.8) p<0.001**	**2.4 (0.2, 4.6) p=0.03**	-0.6 (-1.7, 0.6) p=0.38
EKFC$_{creatinine}$	**3.3 (1.7, 4.9) p<0.001**	0.8 (-2.4, 4.0) p=0.667	-2.2 (-4.7, 0.4) p=0.10
EKFC$_{cystatin}$	**4.0 (0.6, 7.4) p=0.02**	**1.5 (0.2, 2.8) p=0.02**	-1.5 (-3.7, 0.7) p=0.20
EKFC$_{creatinine\text{-}cystatin}$	**7.1 (4.5, 9.7) p<0.001**	**4.6 (2.2, 7.0) p<0.001**	**1.6 (0.2, 3.0) p=0.02**

The table shows for each equation (test B) the difference (95% CI) for percentage of individuals having a change (slope) per year within $\pm$3 mL/min/1.73 m^2 of change in measured GFR minus the equivalent percentage for the CKD-EPI equations (test A), together with a p-value. McNemar's test was used to compare the values of the equations against each other. Positive values indicate that test B is better at monitoring measured GFR over time than test A. Negative values indicate that test A is better at monitoring measured GFR over time than test B. Significant positive differences (p<0.05) shown in bold.

Supplementary Table 7. Comparison of GFR estimating equation's relative performance (rows, test B) over time compared to each of the CKD-EPI equations (columns, test A).

**Test B**	**Test A**		
	$CKD\text{-}EPI_{creatinine}$	$CKD\text{-}EPI_{cystatin}$	$CKD\text{-}EPI_{creatinine\text{-}cystatin}$
$CKD\text{-}EPI_{creatinine}$			
$CKD\text{-}EPI_{cystatin}$	2.3 (-1.6, 6.1)<br>p=0.25		
$CKD\text{-}EPI_{creatinine\text{-}cystatin}$	**6.4 (3.4, 9.4)<br>p<0.001**	**4.1 (1.4, 6.9)<br>p=0.003**	
$CKD\text{-}EPI(2021)_{creatinine}$	0.1 (-0.6, 0.8)<br>p>0.99	-2.2 (-6.0, 1.7)<br>p=0.28	-6.3 (-9.2, -3.3)<br>p<0.001
$CKD\text{-}EPI(2021)_{creatinine\text{-}cystatin}$	**6.3 (3.2, 9.4)<br>p<0.001**	**4.0 (1.4, 6.6)<br>p=0.002**	-0.1 (-1.1, 0.9)<br>p>0.99
$EKFC_{creatinine}$	**2.9 (1.6, 4.2)<br>p<0.001**	0.6 (-3.2, 4.4)<br>p=0.81	-3.5 (-6.5, -0.6)<br>p=0.02
$EKFC_{cystatin}$	**7.4 (3.7, 11.1)<br>p<0.001**	**5.1 (3.1, 7.1)<br>p<0.001**	1.0 (-1.7, 3.7)<br>p=0.49
$EKFC_{creatinine\text{-}cystatin}$	**10.1 (7.1, 13.0)<br>p<0.001**	**7.8 (4.9, 10.6)<br>p<0.001**	**3.7 (1.7, 5.6)<br>p<0.001**

The table shows for each equation (test B) the difference (95% CI) for percentage of individuals having a change (slope) per year within $\leq\pm$5% mL/min/1.73 m^2 of change in measured GFR minus the equivalent percentage for the CKD-EPI equations (test A), together with a p-value. McNemar's test was used to compare the values of the equations against each other.

Positive values indicate that test B is better at monitoring mGFR over time than test A. Negative values indicate that test A is better at monitoring mGFR over time than test B. Significant positive differences (p<0.05) shown in bold.

Supplementary Table 8. Performance of GFR equations: change (slope) per year within ±3 mL/min/1.73 m^2 of slope change of measured GFR in males and females.

Equation	Difference in change per year (eGFR - mGFR) within 3 mL/min/1.73 m^2			
	Males (n=505)		Females (n=370)	
	n/N	%, (95% CI)	n/N	%, (95% CI)
CKD-EPI$_{creatinine}$	371/505	73.5 (69.4, 77.3)	269/370	72.7 (67.9, 77.2)
CKD-EPI$_{cystatin}$	400/505	79.2 (75.4, 82.7)	262/370	70.8 (65.9, 75.4)
CKD-EPI$_{creatinine-cystatin}$	411/505	81.4 (77.7, 84.7)	277/370	74.9 (70.1, 79.2)
CKD-EPI(2021)$_{creatinine}$	369/505	73.1 (69.0, 76.9)	266/370	71.9 (67.0, 76.4)
CKD-EPI(2021)$_{creatinine-cystatin}$	406/505	80.4 (76.7, 83.8)	277/370	74.9 (70.1, 79.2)
EKFC$_{creatinine}$	389/505	77.0 (73.1, 80.6)	280/370	75.7 (71.0, 80.0)
EKFC$_{cystatin}$	407/505	80.6 (76.9, 84.0)	268/370	72.4 (67.6, 76.9)
EKFC$_{creatinine-cystatin}$	413/505	81.8 (78.1, 85.1)	289/370	78.1 (73.5, 82.2)

Supplementary Table 9. Performance of GFR equations: change (slope) per year within ±3 mL/min/1.73 m² of slope change of measured GFR. Sensitivity analysis utilising additional estimated and measured GFR data and modelling approaches

Equation	Difference in change per year (eGFR - mGFR) within ±3 mL/min/1.73 m²					
	Observed difference in change per year (from Table 3, main paper)		Sensitivity analysis 1		Sensitivity analysis 2	
	n/N	% (95% CI)	n/N	% (95% CI)	n/N	% (95% CI)
CKD-EPI$_{creatinine}$	640/875	73.1 (70.1, 76.1)	673/875	76.9 (74.0, 79.7)	704/875	80.5 (77.7, 83.0)
CKD-EPI$_{cystatin}$	662/875	75.7 (72.7, 78.5)	655/875	74.9 (71.8, 77.7)	694/875	79.3 (76.5, 82.0)
CKD-EPI$_{creatinine\text{-}cystatin}$	688/875	78.6 (75.8, 81.3)	685/875	78.3 (75.4, 81.0)	726/875	83.0 (80.3, 85.4)

Sensitivity analysis 1: change in estimated GFR (change per year estimated from linear regression models; one model fitted per person using all available measurements) was compared to observed change in measured GFR (calculated as change per year from difference between 3 year and baseline measurements).

Sensitivity analysis 2: change in estimated GFR (change per year estimated from linear regression model; one model fitted per person using all available measurements) was compared to change in measured GFR (estimated from a multilevel linear regression model using all available measures).

References

1. Turin TC, Tonelli M, Manns BJ, et al. Chronic kidney disease and life expectancy. *Nephrol Dial Transplant* 2012;27:3182-6. doi: 10.1093/ndt/gfs052 [published Online First: 2012/03/24]
2. Bellomo R, Ronco C, Kellum JA, Mehta RL, Palevsky P. Acute renal failure - definition, outcome measures, animal models, fluid therapy and information technology needs: the Second International Consensus Conference of the Acute Dialysis Quality Initiative (ADQI) Group. *Crit Care* 2004;8:R204-12.
3. Levey AS, Coresh J, Greene T, et al. Using standardized serum creatinine values in the modification of diet in renal disease study equation for estimating glomerular filtration rate. *Ann Intern Med* 2006;145:247-54.
4. Rowe C, Sitch AJ, Barratt J, et al. Biological variation of measured and estimated glomerular filtration rate in patients with chronic kidney disease. *Kidney Int* 2019;96:429-35. doi: 10.1016/j.kint.2019.02.021 [published Online First: 2019/05/16]
5. Brochner-Mortensen J. A simple method for the determination of glomerular filtration rate. *Scand J Clin Lab Invest* 1972;30:271-4.
6. Levey AS, Stevens LA, Schmid CH, et al. A new equation to estimate glomerular filtration rate. *Ann Intern Med* 2009;150:604-12.
7. Inker LA, Schmid CH, Tighiouart H, et al. Estimating glomerular filtration rate from serum creatinine and cystatin C. *N Engl J Med* 2012;367:20-9. doi: 10.1056/NEJMoa1114248 [published Online First: 2012/07/06]
8. Pottel H, Bjork J, Courbebaisse M, et al. Development and Validation of a Modified Full Age Spectrum Creatinine-Based Equation to Estimate Glomerular Filtration Rate : A Cross-sectional Analysis of Pooled Data. *Ann Intern Med* 2021;174:183-91. doi: 10.7326/M20-4366 [published Online First: 2020/11/10]
9. Pottel H, Bjork J, Rule AD, et al. Cystatin C-Based Equation to Estimate GFR without the Inclusion of Race and Sex. *N Engl J Med* 2023;388:333-43. doi: 10.1056/NEJMoa2203769 [published Online First: 2023/02/01]
10. Inker LA, Eneanya ND, Coresh J, et al. New Creatinine- and Cystatin C-Based Equations to Estimate GFR without Race. *N Engl J Med* 2021;385:1737-49. doi: 10.1056/NEJMoa2102953 [published Online First: 2021/09/24]
11. Lamb EJ, Barratt J, Brettell EA, et al. Accuracy of glomerular filtration rate estimation using creatinine and cystatin C for identifying and monitoring moderate chronic kidney disease: the eGFR-C study. *Health Technol Assess* 2024;28:1-169. doi: 10.3310/HYHN1078 [published Online First: 2024/07/26]
12. Lamb EJ, Barratt J, Brettell EA, et al. Test accuracy of glomerular filtration rate estimation with creatinine and cystatin C in adults with moderate chronic kidney disease: prospective cohort study. *BMJ Med* 2026;5:e001827. doi: 10.1136/bmjmed-2025-001827 [published Online First: 2026/01/26]
